# Catalytic and Inhibitory Kinetic Behavior of Horseradish Peroxidase on the Electrode Surface

**DOI:** 10.3390/s121114556

**Published:** 2012-10-29

**Authors:** Jitao Huang, Wei Huang, Titi Wang

**Affiliations:** Department of Chemistry and State Key Laboratory of Elemento-Organic Chemistry, College of Chemistry, Nankai University, Tianjin 300071, China

**Keywords:** enzymatic kinetics, inhibition, anomalous behavior, electrochemical biosensor

## Abstract

Enzymatic biosensors are often used to detect trace levels of some specific substance. An alternative methodology is applied for enzymatic assays, in which the electrocatalytic kinetic behavior of enzymes is monitored by measuring the faradaic current for a variety of substrate and inhibitor concentrations. Here we examine a steady-state and pre-steady-state reduction of H_2_O_2_ on the horseradish peroxidase electrode. The results indicate the substrate-concentration dependence of the steady-state current strictly obeys Michaelis-Menten kinetics rules; in other cases there is ambiguity, whereby he inhibitor-concentration dependence of the steady-state current has a discontinuity under moderate concentration conditions. For pre-steady-state phases, both catalysis and inhibition show an abrupt change of the output current. These anomalous phenomena are universal and there might be an underlying biochemical or electrochemical rationale.

## 1. Introduction

Enzymatic electrodes are not only used to detect trace levels of specific substances, but also used to assay enzyme activities. Peroxidases participate in numerous physiological processes, such as cell wall synthesis, plant defence and activation of oxygen. Horseradish peroxidase (HRP) is a representative peroxidase; the mechanisms of peroxidase-catalyzed reactions have been widely studied using HRP, especially in understanding the biological behavior of the catalyzed oxidation of analytes by H_2_O_2_[1–4]. Many H_2_O_2_ biosensors have been constructed using HRP due to its ready availability in high purity and low cost [5–7]. The amperometric biosensors with various enzymes are very useful for the determination of analytes with a very low concentration [8–15]. Enzymatic sensors based on electrochemical reduction of H_2_O_2_[16–18] and oxidation of NADH [19–21] are now available for clinical and environmental detection of glucose, pyruvate, hypoxanthine, alcohol and formaldehyde.

The use of the rotating disk electrode for the study of an electrochemical mechanism was derived by Levich [22]. The output currents obtained are elucidated using the Koutecky-Levich formalism and the ping-pong kinetic scheme for peroxidase [23–25]. In recent years, a large body of research has focused on the mathematical modeling of enzymatic biosensors. Baronas and coworkers [26,27] presented a two-dimensional-in-space mathematical model of amperometric biosensors. They developed later a model based on non-stationary diffusion equations containing a non-linear term related to the Michaelis-Menten kinetics of amperometric enzyme electrodes [28]. Subsequently, they also provided another model of the biosensor based on the mixed enzyme kinetics and diffusion limitations in the case of substrate inhibition [29], the system of non-linear reaction-diffusion equations [30], and the synergistic substrate determination [31]. Andreu and coworkers [32] formulated analytical expressions describing the voltammetric response of a reagentless mediated enzyme electrode operated under rotating disk conditions. Patre and Sangam [33] built a model based on a diffusion mechanism related to Michaelis-Menten kinetics, which can be used in a membrane of the biosensor. Loghambal and Rajendran [34] worked out a steady-state non-linear reaction/diffusion equation. They also developed a mathematical model of amperometric and potentiometric biosensor [35]. Recently, Garay and coworkers [36] presented a comprehensive numerical treatment of the diffusion and reactions within a sandwich-type amperometric biosensor.

In our work, the electrochemical behavior of HRP enzyme activity is reexamined using H_2_O_2_ reduction on the electrode. This enzymatic biosensor is optimized for the detection of HRP activity, including the catalytic and inhibitory kinetic behaviors in steady-state and pre- steady-state phases. Actually, only enzymatic activity during steady state follows the Michaelis-Menten formalism, and anomalous behaviors could be observed in all other kinetic processes, suggesting that there might be an unknown mechanism for the regulation of mass transfer or/and kinetic rate.

## 2. Experimental

### 2.1. Reagents

Horseradish peroxidase (EC 1.11.1.7, 300 U/mg) was purchased from Aladdin Reagent Co. Ltd (Beijing, China). Bovine serum albumin (BSA) was obtained from NewProbe Chemicals Co. (Beijing, China). Hydrogen peroxide (30%, w/v solution) was purchased from Yingdaxigui Reagent Company (Tianjin, China) and the concentration of the more diluted hydrogen peroxide solutions prepared from 30% hydrogen peroxide was determined by titration with potassium permanganate. Phenylhydrazine was obtained from Sinopharm Chemical Reagent Co. Ltd (Beijing, China). Phosphate buffer solution (PBS, pH 7.0) contained 3.8 M KH_2_PO_4_ and 6.2 M Na_2_HPO_4_. All other reagents were of analytical grade.

### 2.2. Electrode Modification

Prior to modification, the glassy carbon electrode surface was polished with 0.05 mm alumina paste on a micro-cloth and subsequently ultrasonically cleaned thoroughly with acetone, NaOH (1:1), HNO_3_ (1:1), and doubly distilled water and then dried at room temperature. A mixed solution of 2.0 U/mL HRP and 0.5% (w/w) BSA was prepared by dissolving HRP and BSA in 0.2 mL PBS (50 mM, pH 7.0). A volume of 3 mL of the mixed solution was then added dropwise on the surface of the glassy carbon electrode. The surface area of the electrode is 0.28 cm^2^. The HRP/BSA electrode was cross-linked by placing the electrode in a closed vessel contained 25% glutaraldehyde and water vapor for 20 min and dried at room temperature for 1 h and stored at 4 °C until use.

### 2.3. Apparatus

Cyclic voltammetry and amperometric experiments were performed with a LabChem 10M electrochemical workstation (Tianjin, China) and a conventional three-electrode system. The modified glassy carbon electrode served as working electrode, a platinum wire as counter electrode, and a saturated calomel electrode (SCE) as reference electrode. A 0.10 M pH 7.0 phosphate buffer solution (PBS) was used as supporting electrolyte. A magnetic stirrer (approximately 400 rpm) was employed during the amperometric measurements. All experiments were carried out at room temperature.

### 2.4. Cyclic Voltammetry Test

The electrochemical properties of the electrode modified were characterized by cyclic voltammetry. Electrochemical experiments were performed in a conventional electrochemical cell containing a three electrode arrangement and the potential swept from −0.4 to 0.2 V (*vs.* SCE) at a sweeping rate of 0.1 V/s. The cyclic voltammetry experiment was performed in 5 mL pH 7.0 PBS. All experiments were conducted at 25 °C.

### 2.5. Amperometric Assay

Electrochemical experiments were undertaken in the electrochemical cell containing 25 mL of the supporting electrolyte. Amperometric measurements were carried out in a stirred solution (approx. 400 rpm). The electrodes were immersed into a stirred phosphate buffer solution (pH 6.5) containing 1.0 mM hydroquinone as the electron mediator and an initial baseline current was recorded. 0.1 mM H_2_O_2_ solution was added to get an increased steady-state current record. Subsequently, solution of increasing phenylhydrazine concentration was added with a micropipette in succession and the current decrease was recorded. All experiments were conducted at 25 °C.

### 2.6. Correlation Analysis

Pearson correlation was a measurement of the strength and direction of a linear relationship between two groups of data. Statistical significance is determined for a *p*-value <0.05 for all tests. Strong correlation is determined for an absolute *r* value >0.7; weak correlation > 0.5. The bi-variate correlation analysis was carried out by the R statistical package (version 2.13.0; http://www.r-project.org/) [37]. The linear regression analysis was performed by the online Free Statistics and Forecasting Software (version 1.1.23-r6; http://www.wessa.net/slr.wasp).

## 3. Results and Discussion

### 3.1. The Model

The following reaction sequence can be proposed for the case of mediated electrocatalytic reduction of H_2_O_2_ at HRP electrode [38,39]:

(1a) $$\text{HRP}({\text{Fe}}^{3+})+{\text{H}}_{2}{\text{O}}_{2}\overset{{\text{k}}_{1}}{⟶}\text{Compound}(\text{I})+{\text{H}}_{2}\text{O}$$

(1b) $$\text{Compound}(\text{I})+{\text{QH}}_{2}\overset{{\text{k}}_{2}}{⟶}\text{Compound}(\text{II})+\text{Q}$$

(1c) $$\text{Compound}(\text{II})+{\text{QH}}_{2}\overset{{\text{k}}_{3}}{⟶}\text{HRP}({\text{Fe}}^{3+})+\text{Q}$$

(1d) $$\text{Q}+2\text{e}\overset{{\text{k}}_{4}}{⟶}{\text{QH}}_{2}$$

where HRP is horseradish peroxidase. Fe^3+^ is the HRP cofactor. Compound (I) (oxidation state +5) and Compound(II) (oxidation state +4) are oxidized intermediates. QH_2_ and Q represent hydroquinone and its oxidized form (benzoquinone), respectively. In these reactions, a two-electron oxidation of the ferriheme moiety of HRP enzyme is caused by H_2_O_2_. The resulting intermediate Compound (I) consists of oxyferryl iron and a porphyrin π cation radical. Compound (I) is reduced to Compound (II) and then it was further reduced to the original form of HRP (Fe^3+^) by the mediator QH_2_. Q is finally reduced back to QH_2_ by a rapid reaction involving the acceptance of two electrons from the electrode.

The inhibition of phenylhydrazine to HRP is an anticompetitive inhibition [39]. When the inhibitor (phenylhydrazine) is added into the electrolyte solution, it can combine with Compound (I) causing the decrease of the current *i*. The possible mechanism of inhibition could be represented as follows:

(2) $$\begin{array}{c} \text{HRP}({\text{Fe}}^{3+})+{\text{H}}_{2}{\text{O}}_{2}\overset{{\text{k}}_{1}}{⟶}\text{Compound}(\text{I})\overset{{\text{k}}_{2}}{⟶}\ldots \\ +\\ \text{I}\\ ⥮{\text{k}}_{1}\\ \text{Compound}(\text{I})‐\text{I} \end{array}$$

where compound(I) - I represents complex of compound(I) with inhibitor.

After adding substrate or inhibitor dropwise, the current rapidly returns toward equilibrium (steady state) with increasing time. For the pre-steady-state part of enzymatic reaction, the current obeys exponential decay equation, *i.e.*,

(3) $$\text{i}={\text{i}}_{\infty }+\text{A}\times \exp(-\text{t}/\tau )$$

where *i* is the faradaic current at time *t. i*_∞_ is the steady-state current. τ is relaxation time, being equal to the time constant of an exponential return of a faradaic current to equilibrium after adding substrate or inhibitor, which reflects the rate at which excited current return to steady-state current.

Suppose that a steady-state current (*i*_∞_) is the current when the biosensor system approaches to equilibrium as *t* → ∞:

(4) $${\text{i}}_{\infty }=\underset{\text{t}\to \infty }{\lim}\text{i}(\text{t})$$

where *i*(*t*) is the current at time *t*. In practice, the current at the response time *t*_R_ is assumed as *i*_∞_. *t*_R_ is defined as the time when the absolute current slope falls below a given small value (ε < 0.0001) [27], *i.e.*,

(5) $${\text{t}}_{R}=\underset{\text{i}>0}{\lim}\left\{\text{t}:\frac{1}{\text{i}(\text{t})}\left|\frac{\text{di}(\text{t})}{\text{dt}}\right|<ɛ\right\}$$

A reduction current (*i*_∞_) observed on electrode is a combination of the mass transport-limited current (*i*_∞,L_) and the reaction-rate-limited current (*i*_∞,K_) [40], accords with the Koutecky-Levich equation, *i.e.*,

(6) $$\frac{1}{{\text{i}}_{\infty }}=\frac{1}{{\text{i}}_{\infty ,\text{L}}}+\frac{1}{{\text{i}}_{\infty ,\text{K}}}$$

For a rotating disk electrode, the mass-transport-limited current (*i*_∞,L_) depends on the angular rotation velocity (ω) of the electrode and the bulk concentration ([H_2_O_2_]) of H_2_O_2_, *i.e.*, *i*_∞,L_ is determined by the Levich equation [22,41]:

(7) $${\text{i}}_{\infty ,\text{L}}=0.62\mathrm{nA}\text{F}AD^{2/3}\nu^{-1/6}\omega^{1/2}[{\text{H}}_{2}{\text{Q}}_{2}]$$

where *n* is the number of electrons transferred to the enzyme in one catalytic cycle (*n* = 2). F is the Faraday constant. *A* is the area of the electrode surface. *D* is the diffusion coefficient of H_2_O_2_. ν is the kinematic viscosity of water. For a given biosensor that is rotating at a constant angular velocity, *i*_∞,L_ is proportional to [H_2_O_2_], Equation (7) reduces to:

(8) $${\text{i}}_{\infty ,\text{L}}={\text{a}}_{1}[{\text{H}}_{2}{\text{Q}}_{2}]$$

where *a*_1_ is constant (*a*_1_ = 0.62*n*F*AD*^2/3^ν^−1/6^ω^1/2^).

When the transport of H_2_O_2_ is high enough to keep its concentration at the electrode surface equal to that in the bulk solution, the reaction-rate-limited current *i*_∞,K_ is governed by the ping-pong kinetic scheme, being expressed by [24,39]:

(9) $$\frac{1}{{\text{i}}_{\infty ,\text{K}}}=\frac{1}{nF\mathrm{A\Gamma}}(\frac{1}{{\text{k}}_{1}[{\text{H}}_{2}{\text{Q}}_{2}]}+\frac{\left[\text{I}\right]}{{\text{k}}_{\text{I}}}+\frac{{\text{k}}_{2}+{\text{k}}_{3}}{{\text{k}}_{2}{\text{k}}_{3}}\frac{1}{\left[{\text{QH}}_{2}\right]}+1)$$

Where *Γ* is the surface concentration of HRP enzyme. [QH_2_] is the concentration of hydroquinone. [I] is the concentration of phenylhydrazine inhibitor. Assuming that *a*_2_ = 1/*nFAΓk*_1_, *b* = 1/*nFAΓk*_I_, and *c*_1_ = (*k*_2_ + *k*_3_)/(*n*F*AΓk*_2_*k*_3_[QH_2_])*+*1/*n*F*AΓ*, Equation (9) can be also reduced to:

(10) $$\frac{1}{{\text{i}}_{\infty ,\text{K}}}={\text{a}}_{2}\frac{1}{\left[{\text{H}}_{2}{\text{O}}_{2}\right]}+\text{b}[\text{I}]+{\text{c}}_{1}$$

Substituting Equations (8) and (10) into Equation (6), the Koutecky-Levich equation can be reduced to:

(11) $$\frac{1}{{\text{i}}_{\infty }}={\text{a}}_{3}\frac{1}{\left[{\text{H}}_{2}{\text{O}}_{2}\right]}+\text{b}[\text{I}]+{\text{c}}_{1}$$

where *a*_3_ = *a*_2_ + 1/*a*_1_.

Combining *b*[I] term with constant *c*_1_ in Equation (11) in a constant phenylhydrazine concentration, we obtain *c*_2_ = *c*_1_ + *b*[I]. The dependence of the steady-state current on the substrate concentration is written as:

(12) $$\frac{1}{{\text{i}}_{\infty }}={\text{a}}_{3}\frac{1}{\left[{\text{H}}_{2}{\text{O}}_{2}\right]}+{\text{c}}_{2}$$

Combining *a*_3_/[H_2_O_2_] term with constant *c*_1_ in Equation (11) in a constant H_2_O_2_ concentration, we obtain *c*_3_ = *c*_1_ + *a*_3_/[H_2_O_2_]. The dependence of the steady-state current on the inhibitor concentration is written as:

(13) $$\frac{1}{{\text{i}}_{\infty }}=\text{b}[\text{I}]+{\text{c}}_{3}$$

### 3.2. Electrochemical Biosensor

Horseradish peroxidase (HRP) and bovine serum albumin (BSA) are immobilized on the surface of a glassy carbon (GC) electrode. Hydroquinone/quinone mediator facilitates the electron transfer between protein and the electrode. Experiments are performed with H_2_O_2_ to ascertain if HRP is indeed immobilized on the electrode.

**Figure 1.**
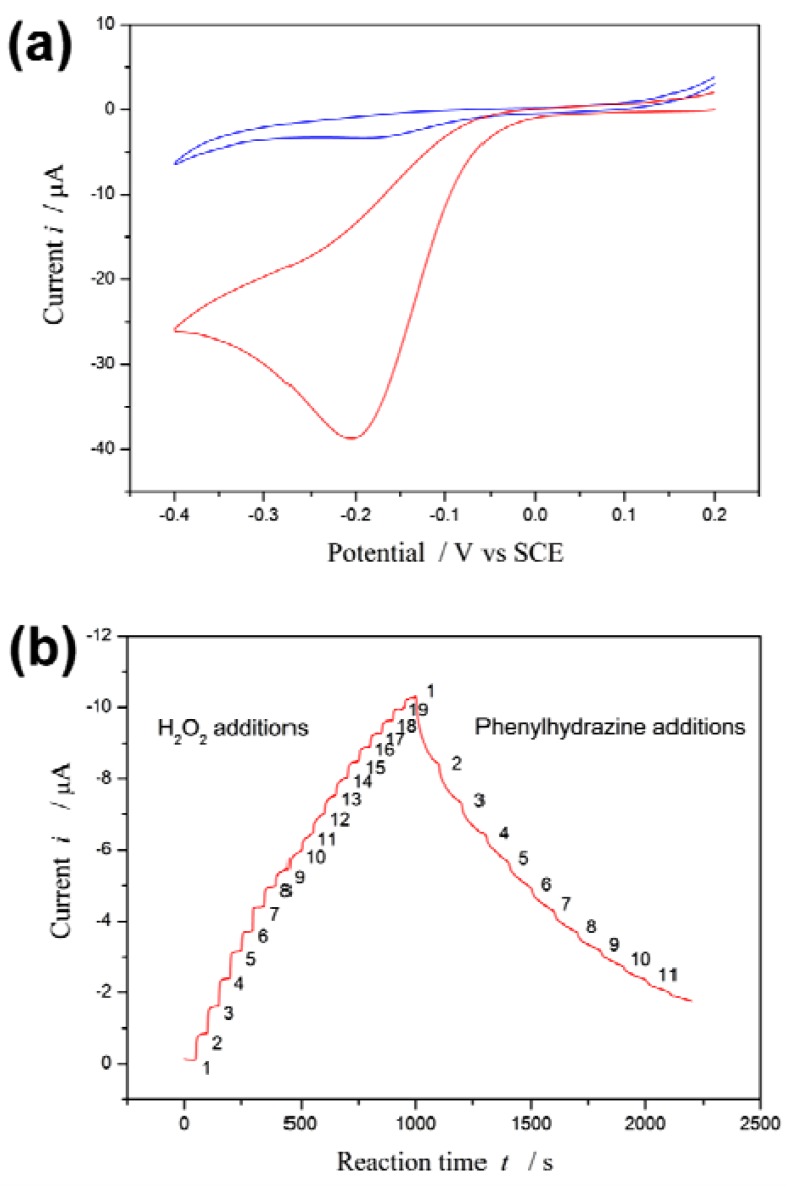
(**a**) Cyclic voltammogram of the HRP/BSA biosensor in PBS buffer (pH 7.0): in the absence (blue curve) and presence (red curve) of 3.2 mM H_2_O_2_. Scan rate = 0.1 V/s. (**b**) Current-time response of the HRP/BSA electrode on 19 successive titrations of H_2_O_2_ (0.1 mM each time) and subsequently 11 titrations of phenylhydrazine (0.5 mM each time) into the stirring electrolyte solution. Applied potential: −0.2 V.

Figure 1(a) depicts the cyclic voltammetry curves of the proposed biosensor in the presence and absence of H_2_O_2_. A redox couple is observed in the absence of H_2_O_2_. However, an enhancement of the peak current is observed in the presence of H_2_O_2_. On the other hand, it has been reported that phenylhydrazine is able to inhibit or abrogate HRP activity by competing with the normal hydroquinone [42,43]. The inhibition effect of phenylhydrazine is measured by using the proposed biosensor.

Upon 19 successive additions of 0.1 mM H_2_O_2_ substrate to the electrolyte solution, *i* increases with [H_2_O_2_]. Subsequently, upon 11 successive additions of 0.5 mM phenylhydrazine inhibitor to the electrolyte solution, *i* decreases with increasing [I] see Figure 1(b).

The steep increased (or decreased) in magnitude of the current is observed when H_2_O_2_ (or phenylhydrazine) is added into the electrolyte solution, and then the current returns to equilibrium (steady state *i*_∞_) following an exponential decay with a certain recovery speed (relaxation time τ). The values of *i*_∞_ and τ are best computed by non-linear regression of data fitted to Equation (2). These values at different substrate concentration are listed in Table S1 (Appendix); they at different inhibitor concentration in Table S2 (Appendix).

### 3.3. Electrochemical Enzyme Assay

**Figure 2.**
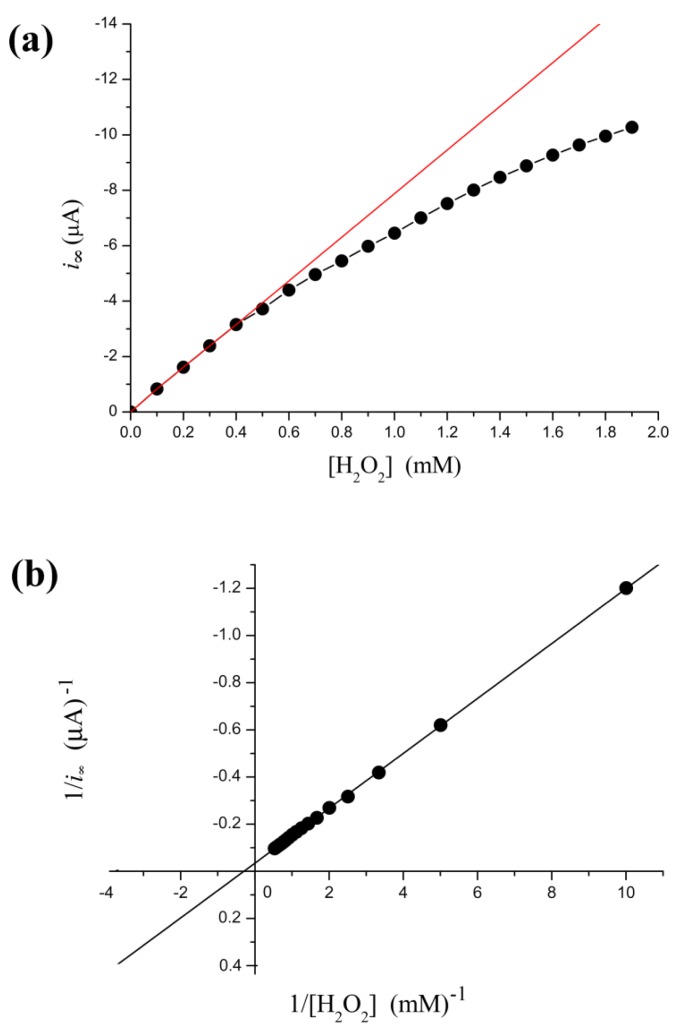
Effect of the concentrations of H_2_O_2_ substrate on the steady-state current. (**a**) Lineweaver-Burk plot. (**b**) Michaelis-Menten plot. The initial four points are fitted by a calibration line (red line) with the slopes of −7.86 and intercept of −0.025 (*r* = −0.999; *p* < 0.0001).

From Figure 2(a), the relation between *i*_∞_ and [H_2_O_2_] is not linear. The calibration curve is valid only under low concentration of substrate conditions. More specifically, when [H_2_O_2_] < 0.4 mM, the relation is very close to a straight line (calibration curve): *i*_∞_ = −7.86(±0.074) × [H_2_O_2_] − 0.025(±0.018) with *r* = −0.999; *p* < 0.0001. This linear correlation is a prerequisite for applying the electrochemical biosensors. Thus electrocatalysis reduction to H_2_O_2_ with HRP electrode can detect trace levels of H_2_O_2_. In fact, our result shows the steady-state current follows the Michaelis-Menten kinetic model within a broader concentration range. Kinetic constants are determined by fitting the initial rate data to Lineweaver-Burk plot Figure 2(b). Thus, the current is regarded as a relative enzyme reaction rate. This methodology could monitor in real-time subtle changes in enzyme activity.

**Figure 3.**
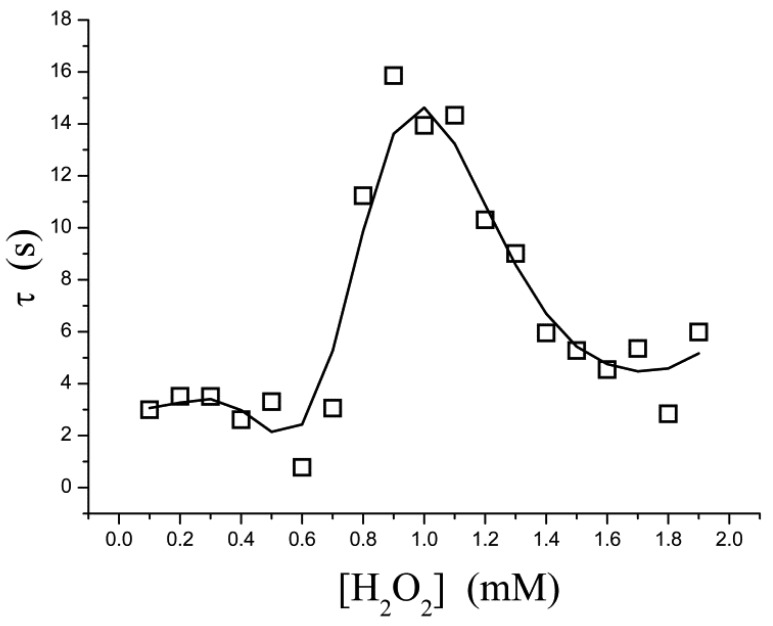
Effect of the concentrations of H_2_O_2_ substrate on the relaxation time during pre-steady-state phase.

The pre-steady-state time course of the *i* → *i*_∞_ process is fitted to Equation (3) for a single exponential decay, in which τ is the apparent relaxation time for output current (*i.e.*, the reciprocal of the apparent first-order rate constant). It is found, however, that τ is not a simple function of the substrate concentration. As shown in Figure 3, τ is low in both the low- and high-concentration regions; a relatively larger τ occurs in at a moderate concentration ([H_2_O_2_] = 0.8–1.4 mM). This suggests that, within this concentration range, the rate at which the equilibrium is approached is slowed down.

If the kinetic control becomes operative, there is a possible explanation: a “shoulder” appears on the curve of *t*_0.5_*versus*[H_2_O_2_] in the two-dimensional-in-space biosensor model, as has been proposed in reference [27]. Where *t*_0.5_ is the half time (*t*_0.5_ = 0.693τ). Baronas *et al.*[27] showed that the shoulder occurs at [H_2_O_2_] near to the Michaelis constant *K*_m_. At [H_2_O_2_] ≪ *K*_m_ (τ = 1/*k*_1_*K*_m_) the reaction kinetics for [H_2_O_2_] is a zero order throughout the biosensor, whereas for [H_2_O_2_] ≫ *K*_m_ (τ = 1/*k*_1_[H_2_O_2_]) the kinetics is a first order throughout. At intermediate values of [H_2_O_2_] the kinetics undergoes a transition from zero order to first order. This phenomenon is also manifested in inhibition assays, so it is not obvious whether agreement between our data and the shoulder of *t*_0.5_ is not a coincidence. Inhibition kinetics also experiences a transition from zero order to first order, but it is not proof.

### 3.4. Electrochemical Inhibition Assay

**Figure 4.**
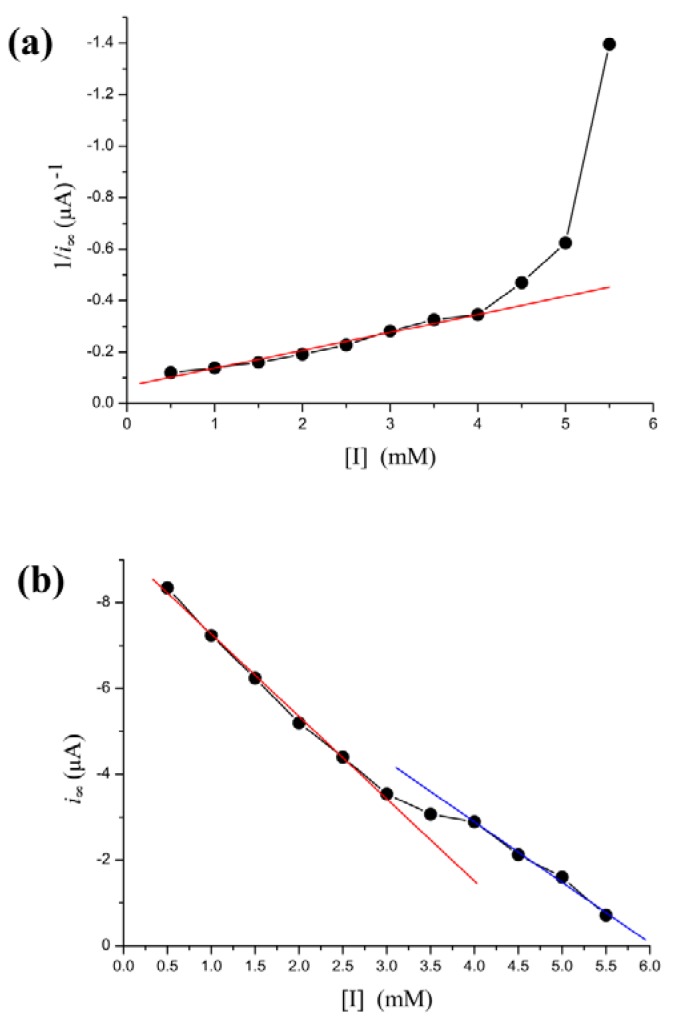
Effect of the concentrations of phenylhydrazine inhibitor on the steady-state current. (**a**) Lineweaver-Burk plot. (**b**) The relationship between *i*_∞_ and [I]. The initial seven points are fitted by a straight line with a slope of −1.92 and intercept of −9.19 (*r* = −0.999; *p* < 0.0001). The final four points are fitted by a straight line with a slope of −1.41 and intercept of −8.54 (*r* = −0.999; *p* < 0.0001). Discontinuity occurs at [I] = 3–4 mM.

Phenylhydrazine can inhibit HRP activity [42,43]. In our biosensor, *i*_∞_ decreased steadily as [I] is increased. However, the Lineweaver-Burk plot for the substrate dependence shows that the reaction does not follow Michaelis-Menten kinetics, as the curve connecting the experimental points is clearly curved Figure 4(a). However, there is a more simple linear relationship between *i*_∞_ and [I]. The dependence of the rate of *i*_∞_ change on [I] is illustrated in Figure 4(b). As shown in the figure, the regression line is discontinuous at inhibitor concentration of 3–4 mM. The [I]-*i*_∞_ relation is broken into two individual lines. When [I] < 3 mM, decrease of *i*_∞_ along a regression line is given by the equation: *i*_∞_ = −1.92(±0.075) × [I] − 9.19(±0.03) with *r* = −0.999; *p* < 0.0001. When [I] > 4 mM, decline of *i*_∞_ along another line is given by the equation: *i*_∞_ = −1.41(±0.024) × [I] − 8.54(±0.1) with *r* = −0.999; *p* < 0.0001.

**Figure 5.**
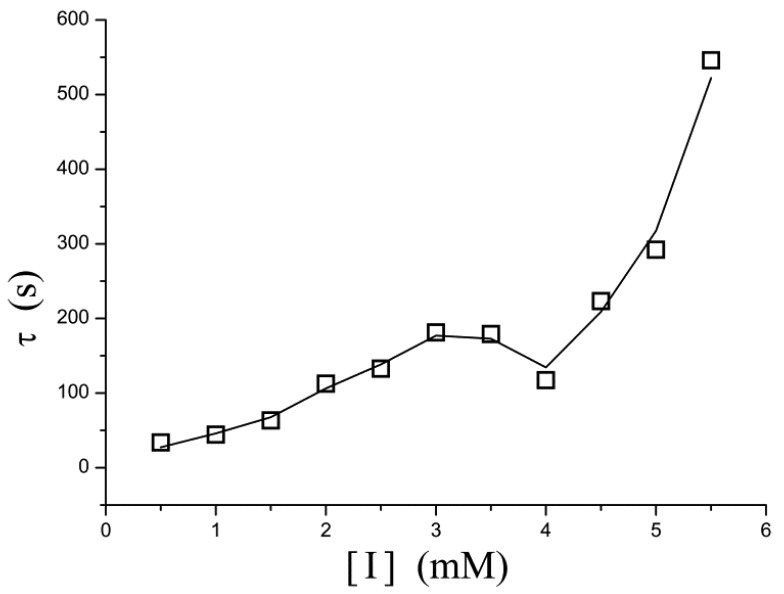
Effect of the concentration of phenylhydrazine inhibitor on the relaxation time during pre-steady-state phase. It increased exponentially with increase in concentration. A “depression” appears on the curve in the concentration of 3–4 mM.

A similar phenomenon occurs in the relation between [I] and τ as well. In Figure 5, τ increases exponentially as the inhibitor concentration increases. However, in the concentration of 3–4 mM, the points determined experimentally is lower than τ value extrapolated from low concentration and thus the curve displays a drop.

## 4. Conclusions

We have examined the electrocatalytic behavior of enzymatic biosensors and possibilities for measuring the enzymatic activities. The substrate concentration-dependence of such a steady-state current is governed by the Michaelis-Menten equation under the condition of constant diffusion, suggesting that the output current reflects the relative rate of the enzyme reaction. For our datasets, we find that Michaelis-Menten model does not work well at high inhibitor concentrations and works better at low concentrations, and the enzymatic kinetics gives the best results in the absence of inhibitor. Deviations from this model cause observed sudden changes in the current occurring at intermediate concentrations of inhibitor or substrate. An auto-deceleration of the current occurs when the substrate reaches a specific concentration ([H_2_O_2_] = 0.7 mM). In contrast, an auto-acceleration occurs when the inhibitor reaches another critical concentration ([I] = 3 mM).

Electrochemical enzyme assays provide an extremely sensitive measure but suffer from difficulties in interpretation of the data. At relatively higher concentrations of H_2_O_2_, the steady-state current follows the Michaelis-Menten equation (Figure 2) and the relaxation time is low (Figure 3). These indicate that no significant H_2_O_2_-induced suicide inactivation of HRP enzyme occurs in our experiment. The Levich equation and ping-pong kinetics emphasize that the current should be a monotonically increasing (or decreasing) function of the concentration of substrate (or inhibitor) either under diffusion limitations or under enzyme kinetics. However, peaks, discontinuities and sudden changes appear in Figures 3–5 and their definite mechanism is unknown yet. Also, it is not clear whether the same would be true for other enzymes. Electrochemical biosensors do not provide any microscopic information, and even assignment of the relaxation modes (whether it is the mode of a mass transfer or of a chemical reaction) is not obvious.

## Appendix

**Table S1.** The steady-state current (*i*_∞_) and pre-steady-state relaxation time (τ) at different substrate concentrations ([H_2_O_2_]).

**[H_2_O_2_]mM**	***i*_∞_(μA)**	**τ (s)**	**Accuracy**	

			**χ^2^/dof**	***r***
0.1	−0.833	2.996	3.01 × 10^−4^	0.990
0.2	−1.614	3.513	4.52 × 10^−4^	0.992
0.3	−2.389	3.507	4.08 × 10^−4^	0.992
0.4	−3.153	2.614	2.20 × 10^−4^	0.993
0.5	−3.718	3.307	9.32 × 10^−5^	0.995
0.6	−4.403	4.370	1.87 × 10^−4^	0.985
0.7	−4.961	3.059	3.17 × 10^−4^	0.979
0.8	−5.450	11.232	5.94 × 10^−4^	0.962
0.9	−5.979	15.859	3.71 × 10^−4^	0.981
1.0	−6.451	13.940	8.40 × 10^−4^	0.964
1.1	−7.006	14.331	6.21 × 10^−4^	0.977
1.2	−7.520	10.307	5.10 × 10^−4^	0.980
1.3	−8.008	9.009	3.47 × 10^−4^	0.984
1.4	−8.468	5.952	2.27 × 10^−4^	0.988
1.5	−8.881	5.278	2.24 × 10^−4^	0.986
1.6	−9.268	4.542	3.17 × 10^−4^	0.973
1.7	−9.633	5.355	1.49 × 10^−4^	0.988
1.8	−9.953	2.840	4.20 × 10^−4^	0.927
1.9	−10.272	5.987	3.87 × 10^−4^	0.954

**Table S2.** The steady-state current (*i*_∞_) and pre-steady-state relaxation time (τ) at different phenylhydrazine inhibitor concentrations ([I]).

**[I] mM**	***i*_∞_(μA)**	**τ (s)**	**Accuracy**	

			**χ^2^/dof**	***r***
0.5	−8.348	33.777	1.48 × 10^−3^	0.997
1.0	−7.235	44.277	6.57 × 10^−4^	0.995
1.5	−6.240	63.354	5.21 × 10^−4^	0.994
2.0	−5.201	112.63	3.84 × 10^−4^	0.994
2.5	−4.402	132.71	4.24 × 10^−4^	0.992
3.0	−3.543	181.32	2.61 × 10^−4^	0.994
3.5	−3.069	179.08	3.42 × 10^−4^	0.990
4.0	−2.893	117.37	2.19 × 10^−4^	0.991
4.5	−2.128	223.28	1.58 × 10^−4^	0.992
5.0	−1.602	292.14	1.71 × 10^−4^	0.989
5.5	−0.716	545.95	1.62 × 10^−4^	0.986
